# Prevalence of syphilis in female sex workers in three countryside cities of the state of Pará, Brazilian Amazon

**DOI:** 10.1186/s12879-020-4850-1

**Published:** 2020-02-11

**Authors:** Ronaldo Lopes de Souza, Lucimar Di Paula dos Santos Madeira, Marcelo Victor Serejo Pereira, Rachel Macedo da Silva, João Bráullio de Luna Sales, Vania Nakauth Azevedo, Rosimar Neris Martins Feitosa, Jacqueline Cortinhas Monteiro, Marluisa de Oliveira Guimarães Ishak, Ricardo Ishak, Andre Luis Ribeiro Ribeiro, Aldemir B. Oliveira-Filho, Luiz Fernando Almeida Machado

**Affiliations:** 1 Virology Laboratory, Institute of Biological Sciences, Federal University of Pará, Brazil. Augusto Correa 1. Guamá. CEP 66075-110, Belém, Pará Brazil; 2 Laboratório de Microbiologia, Instituto de Ciências Biológicas, Universidade Federal do Pará, Belém, Pará Brazil; 3 Laboratório de Virologia, Instituto de Ciências Biológicas, Universidade Federal do Pará, Belém, Pará Brazil; 4 Instituto de Ciências Exatas e Naturais, Universidade Federal do Pará, Belém, Pará Brazil; 5 Laboratorio de Lepidopterologia e Ictiologia Integrada, Centro de Estudos Avançados em Biodiersidade (CEABIO), Universidade Federal do Pará, Belém, Pará Brazil; 6 Grupo de Estudo e Pesquisa em Populações Vulneráveis, Instituto de Estudos Costeiros, Universidade Federal do Pará, Bragança, Pará Brazil

**Keywords:** Syphilis, Sex workers, Amazon region, Epidemiology

## Abstract

**Background:**

Syphilis is a sexually transmitted infection (STI) transmitted from person to person mainly by sexual intercourse or through vertical transmission during pregnancy. Female sex workers (FSWs) are exposed especially to syphilis infection, and besides all the efforts to control the spread of STIs, syphilis prevalence is still rising, mainly occurring in low-income countries. This study aimed to investigate the syphilis prevalence, demographic characteristics and sexual habits among FSWs in the Amazon region of Brazil.

**Methods:**

A cross-sectional study was carried out including 184 FSWs from 3 countryside cities of the state of Pará, Amazon region of Brazil. A venereal disease research laboratory test and an indirect immunoenzyme assay to test antibodies against Treponema pallidum were used for screening syphilis infection, while sexual habits and demographic data information were collected through a semi-structured questionnaire. Data was analyzed comparing groups with/without syphilis. Poisson regression models were used to estimate the reasons of prevalence (RP).

**Results:**

The overall prevalence of syphilis was 14.1% (95% CI = 9.8–17.8). FSWs had between 15 and 56 years of age, most were unmarried (65.7%), had attended less than 8 years of formal education (64.1%), had between 10 and 20 partners per week (64.1%), and reported no previous history of STIs (76.1%) and regular use of condom (52.7%). Low level of education attending up to the primary school (RP adjusted = 3.8; 95% CI = 1.4–9.2) and high frequency of anal sex during the past year (RP adjusted = 9.3; 95% CI = 3.5–28.7) were associated with a higher prevalence of syphilis.

**Conclusions:**

A high prevalence of syphilis among FSWs in the Brazilian Amazon region was identified, showing that syphilis is more likely to be transmitted in FSW working in low-income areas, which is attributed to the low level of education. Anal intercourse was found as a risk factor associated with syphilis. Health programs focused on risk populations appear as a rational way to control syphilis spread, which is a rising problem in Brazil and in other several countries.

## Background

Brazil has an important emerging economy and the largest territorial area in South America, however, the population still live in a wide gap of socioeconomic inequality. This combination of large territorial area (with several remote areas) and economic inequality creates a big challenge to governments to provide social inclusion, access to appropriate education and healthcare [1]. These characteristics are seen in other emerging countries such as Russia, India, China and South Africa (BRICS group), that represent over 3.1 billion people, or about 41% of the world population. These countries have wealthy and developed cities/regions (usually in large metropolitan areas), but also extreme poverty and low human development, which may represent most of their population. Thus, this paper focused on this last group of people, where syphilis is more likely to be endemic.

Syphilis is a sexually transmitted infection (STI) caused by *Treponema pallidum* subspecies *pallidum*, a spirochaete bacterium well-known for its invasiveness and immune-evasiveness [2]. This bacterium is primarily transmitted by sexual exposure or through vertical transmission during pregnancy. Syphilis is still a public health problem worldwide and low-income countries have endemic rates of syphilis among their general populations while middle- or high-income countries have concentrated epidemics of syphilis in specific populations, for example, sex workers [3]. The World Health Organization (WHO) estimates that around 6 million new cases of syphilis occur every year among adults aged between 15 and 49 years [4]. Many factors are associated with higher risks of acquiring an STI, such as syphilis, and female sex workers (FSWs) are particularly exposed to them, however, limited information is available on this group of people often discriminated by many societies.

FSWs are considered a high-risk group of women vulnerable to STIs, such as syphilis and human immunodeficiency virus (HIV)/acquired immunodeficiency syndrome (AIDS) infection [5]. In Brazil, it is estimated that half-million women are involved in sex works, which corresponds to approximately 0.8% of the female population aged between 15 and 49 years in the country [6].

Studies have shown epidemiological heterogeneity on the prevalence of STIs among different regions of Brazil [7, 8]. A survey conducted in 10 different state capital cities in Brazil (the seat of a state government, in this case with an average large metropolitan area > 1.5 million inhabitants) showed an increase from 2.4% in 2009 to 8.5% in 2016 on the prevalence of syphilis among FSWs, which demonstrates that beside all health public policies and access to information available, the prevalence of syphilis is rising [9]. Another study carried out in three southern cities of Brazil (Tubarão, Laguna and Imbituba), showed an even higher prevalence of syphilis among FSWs (19.7%) [8].

Although a few northern cities can eventually be included in national surveys, epidemiological information on STIs, especially syphilis, is still scarce in northern Brazil, which reflects the socioeconomic attention given to this region that is the poorest and less developed in the country. To date, the only epidemiological study focusing on syphilis in FSWs in this region pointed out a high prevalence of *T. pallidum* (37%) associated with the use of illicit drug and having sex without condom [10]. Thus, this study aimed to investigate the seroprevalence of syphilis and risk factors associated with syphilis acquisition among FSWs in three countryside cities of the state of Pará, Brazilian Amazon region.

## Methods

### Study design and ethical aspects

A cross-sectional seroprevalence survey was carried out between January 2007 and June 2008. A convenience (non-probabilistic) sample was used to gather data from participants, which enrollment was carried out three times a week in their workplaces (streets, bars, websites, special strip clubs, and etc) and was determined by a sample size analysis. The study was approved by the Research Ethics Committee Involving Human Beings of the Federal University of Pará, under protocol number 25/2006.

**Fig. 1:**
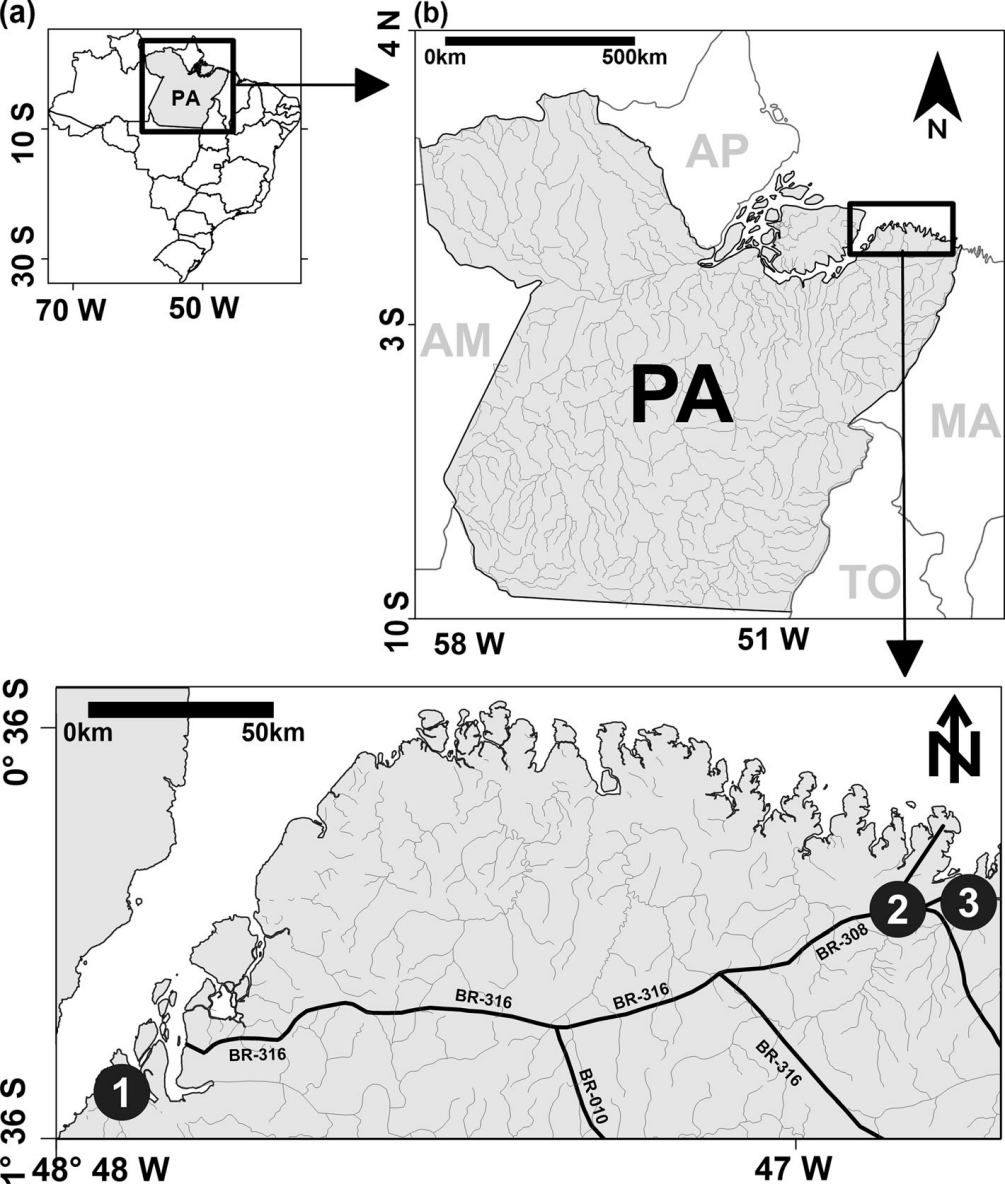
Geographical location of host cities. The three cities where this study took are located in Northern Brazil (**a**), state of Pará (**b**). The location of the cities of Barcarena (1), Bragança (2) and Augusto Corrêa (3) are indicated on the map (**c**)

The sample size determination was based on the estimated prevalence of syphilis in the FSWs population in Brazil (20%) and resulted in a minimum sample size of 180 participants. The sample error (ε) assumed was 5%, and test power of 80% was established. Samples were collected from three countryside cities: Augusto Corrêa, Barcarena and Bragança (Fig. 1). These cities can be characterized as small urban areas (population is between 50,000 and 200,000), showing about 100,000 inhabitants, have human development indexes around 0.55 (low) and have as their main economic activities: fishing, agriculture, extraction and processing of natural resources, such as crabs, shrimps and kaolin (used in the production of alumina and aluminium). All these cities have an intense flow of people and product trade, mainly by highways and seaports [9]. The following inclusion criteria were used: women (biologically determined at birth) older than 15 years who reported commercial sex at least once in the last 3 months that agreed to participate in the study and signed the free consent form. All participants were assured confidentiality.

Participants were invited to answered a questionnaire including socio-demographic data (age, years of study, marital status) and sexual behaviour: condom use and use of alcoholic beverages during sexual service (last 30 days); number of average weekly sexual partners; and if they had performed any of these activities during the last 12 months of sex work: practice and frequency of anal sex, had sex with partners from other Brazilian states, changes in genitalia (occurrence of genital ulcer), history of STIs such as HIV, diagnosis of STIs included history of previous syphilis diagnosis/treatment and the use of illicit drugs. The frequency of anal sex was defined according to the occurrence of this practice in sexual meetings: high (≥50%), sometimes (< 50%) or never.

The length of formal education was divided into two groups: up to primary education, which is equivalent to 8 years of schooling in Brazil, secondary school or more. The number of sexual partners per week was divided into 3 categories based on the observation of the data. The three groups were determined according to the following groups: up to 9, 10 to 20 and more than 20 partners. Marital status was defined as married or unmarried, the last included all women who were single, divorced, separated or widowed.

### Laboratory tests

Blood sample (5 mL) was collected in a vacuum collection tube containing ethylenediaminetetraacetic acid (EDTA) as anticoagulant from each participant. Each participant received a randomized number and all blood samples were processed blindly by a single staff. All samples were frozen and stored at − 20 °C and processed in the Laboratory of Virology of Federal University of Pará, where the non-treponemal test Venereal Disease Research Laboratory (VDRL) test (Biovdrl, Biomerieux) was carried out. The VDRL reagent samples were subjected to an enzyme-linked immunosorbent assay (ELISA) for the detection of anti-treponemal antibodies (BioRich Syphilis®). All reactions were done following the instructions of the manufacturers. Results were considered positive for syphilis when both tests (VDRL and ELISA) were reagent and VDRL results was ≥1:8. All participants were notified about the blood test results and those who tested positive were referred to public health services for treatment and follow-up.

### Statistical analysis

All study data were entered into an Excel database and converted to SPSS file, which was used for all statistical analysis. Ninety-five per cent confidence intervals (95% CI) were determined for syphilis prevalence in total and for each individual city. Positive syphilis test was the main outcome measure. For each category of exploratory variables, reasons of prevalence (RP) were estimated using Poisson regression models, having as a reference the category of least expected risk. Raw prevalence for each exploratory variable was estimated by modeling technique using Wald statistic with significance level of 5%. In a regression model that simultaneously included all variables that presented a value *p* < 0.05, using Wald statistics, the adjusted reasons of prevalence were calculated.

## Results

A total of 184 FSWs living in the cities of Augusto Corrêa (*n* = 32), Barcarena (*n* = 94) and Bragança (*n* = 58), countryside cities of the State of Pará, Northern Brazil, were enrolled (Table 1). Age varied from 15 to 56 years (average 28 years).

**Table 1:** Syphilis prevalence among female sex workers in three countryside cities of the state of Pará, Amazon region of Brazil

Cities	Overall sample		Syphilis				
			Negative		Positive		
	n	%	n	%	n	%	95% CI
Augusto Corrêa	32	17.4	25	78.1	7	21.9	17.6–26.7
Barcarena	94	51.1	83	88.3	11	11.7	7.7–15.8
Bragança	58	31.5	50	86.2	8	13.8	10.1–19.0
Total	184	100	158	85.9	26	14.1	9.8–17.8

Abbreviations: *CI* confidence interval; Syphilis positive, participants who tested positive in both, venereal disease research laboratory and enzyme-linked immunosorbent assay for *Treponema pallidum*

Most women were unmarried, had up to primary education, regularly used condoms during sexual intercourse, had between 10 and 20 partners per week, had already had contacts with partners from other Brazilian states and reported not having a history of STIs, use of illicit drugs, or had anal sex (Table 2). From those who used illicit drugs, most reported use of only non-injecting drugs (95.9% - 70/73), and only three reported injecting drug use. At the time of the interview, all participants reported that they didn’t have any signs or symptoms of active STIs and had drunk alcoholic beverages during the working period in the last 30 days.

**Table 2:** Sexual habits reports and demographic data among female sexual workers in three countryside cities of the state of Pará, Amazon region of Brazil

Characteristics	Overall sample		Syphilis +		Poisson regression	
	n	%	n	%	p	RP (95% CI)
**Age**						
15–25	103	56.0	16	61.5	0.8	1.3 (0.6–2.9)
26–36	69	37.5	9	34.6		0.9 (0.4–2.0)
> 36	12	6.5	1	3.9		Reference
**Marital status**						
Unmarried	142	77.2	24	92.3	0.2	5.1 (0.7–31.8)
Married	28	15.2	1	3.8		Reference
Data not available	14	7.6	1	3.8		
**Length of education**						
Up to primary school	118	64.1	22	84.6	**0.03**	3.1 (1.1–8.7)
Secondary school or more	66	35.9	4	15.4		Reference
**Use of illicit drugs (injectable/non-injectable)**						
Yes	73	39.7	12	46.2	0.7	1.3 (0.6–2.8)
No	111	60.3	14	53.8		Reference
**Condom use**						
Rarely	36	19.6	5	19.2	0.8	1.0 (0.3–5.7)
Sometimes	51	27.7	8	30.8		1.1 (0.4–3.0)
Always	97	52.7	13	50		Reference
**Anal sex**						
High frequency	12	6.5	7	26.9	**0.02**	11.2 (3.6–31.2)
Sometimes	71	38.6	9	34.6		1.3 (0.5–3.2)
Never	101	54.9	10	38.5		Reference
**Alcohol consumption prior or during work period**						
Always	184	100	23	100	**–**	
No	–	–	–	–		
**Number of sexual partners**						
More than 20	13	7.1	2	7.7	0.9	1.1 (0.2–5.5)
10 to 20	118	64.1	17	65.4		1.1 (0.4–2.7)
Up to 9	53	28.8	7	26.9		Reference
**Sexual partners from other Brazilian states**						
Yes	132	71.7	18	69.2	0.5	1.5 (0.6–5.8)
No	37	20.1	3	11.4		Reference
Data not available	15	8.2	5	19.2		
**STI History**						
Yes	44	23.9	10	38.5	0.1	1.9 (0.9–5.0)
No	140	76.1	16	61.5		Reference

Abbreviations: *RP* Reasons of prevalence, *CI* confidence interval; Syphilis +, participants who tested positive in both, venereal disease research laboratory and enzyme-linked immunosorbent assay for *Treponema pallidum*

The overall prevalence of syphilis was 14.1% in this study. The highest prevalence of syphilis was found in the city of Augusto Corrêa (21.9%), followed by Bragança (13.8%) and Barcarena (11.7%) (Table 1). The majority of FSWs who tested positive to syphilis (VDRL ≥1:8 and ELISA reagent) were between 15 and 25 years of age (61.5%), were unmarried (92.3%), had up to primary school education (84.6%) and no illicit drug use (53.8%). With regards to condom use, 50% of FSWs who tested positive claimed they always used a condom so that would be the majority (30.8% said they sometimes used a condom) (Table 2). Raw Poisson regression models demonstrated a higher prevalence of syphilis among FSWs with a low level of schooling (only primary school) and high frequency of anal sex (Table 2). The adjusted Poisson regression model evidenced that the same exposure variables maintained an independent effect on the occurrence of syphilis in FSWs: up to primary school (RP adjusted = 3.8; 95% CI = 1.4–9.2) and having anal sex during > 50% of sexual encounters (RP adjusted = 9.3; 95% CI = 3.5–28.7).

## Discussion

Although Brazil is classified as an upper-middle-income economy, the northern region is the poorest in the country and the economy ranges from low to lower-middle income in most cities, including those where this study took place. This study is one of the first epidemiological reports on syphilis in FSWs in the Brazilian Amazon region. In this region, the Amazon rainforest occupies large areas of the northern Brazil, which causes relative isolation, resulting (or because of) limited infrastructure and public services and low human development [11]. This scenario has a negative impact on sexual behaviour, increasing risk-taking activities [12–14]. Additionally, gender empowerment is an issue on the population of such regions, which are still male-centred, and women are marginalized in a social context. Subsistence is much harder for women, which can be pushed towards sex work and must succumb to male will.

In fact, acquisition and transmission of *T. pallidum* are related to social, economic, cultural and behavioural factors that influence the occurrence of syphilis in the population, especially among FSWs [2]. The high prevalence of syphilis among FSWs has been reported in low-income countries, such as the Dominican Republic (5.1–11.1%), Honduras (1.3–6.0%), Guatemala (1.1–11.8%), El Salvador (2.7–15.0%) and Peru (2.2–4.1%) [15].

The prevalence of syphilis was high (14.1%), which was 65% higher than a recent study that evaluated a similar population of FSWs from 12 Brazilian cities (8.5%) [16], and 3.5 higher than the prevalence found in the city of Botucatu (4%), southeast Brazil [17] using rapid qualitative test and ELISA. These results indicate the disparity on syphilis prevalence among FSWs in Brazil, as well as the need for syphilis control and preventive measures in marginalized populations, such as the observed in this Amazon region and other populations worldwide. This data reinforces the need for specific approaches in order to control syphilis spread in these populations.

Unfavourable socioeconomic conditions, and consequently low living standards, may favour initiation and involvement in sex work, causing a negative influence self-care and vulnerability to STIs, such as syphilis [15, 18, 19]. Recently, unprotected sex has been linked to as an advantage to get higher payment on sex work reported by FSWs, which was associated to high rates of HIV and HBV infections [12, 14].

This study has shown that low educational level and practice of anal sex are independent risk factors for syphilis infection. Schooling is one of the most important variables to measure the socio-economic status and its effects on the health status of a population. We found that FSWs who had attended up to the primary school showed a significantly higher prevalence of syphilis positivity. This may reflect the low overall awareness of these individuals about the risks involved and sex work and measures to prevent STIs [20, 21].

Having anal sex with high frequency (> 50% of sexual encounters) during the last year was another factor that increased the prevalence of syphilis infection. Anal intercourse increases the risk of STIs because of the characteristic of the rectum, which shows a delicate tissue that can be easily damaged, which is aggravated by the limited lubrification, giving access to bacteria and virus to the bloodstream [22–24]. Studies have shown that anal intercourse carries one of the highest-risk of sexual activity for getting HIV. Our results suggest that anal intercourse not only increases the risk of HIV acquisition but also from *T. pallidum*, and the same infection mechanism can be applied to men who have sex with men and heterosexuals [14, 23, 24].

Although several epidemiological studies have indicated anal sex as a risk factor for acquisition and transmission of *T. pallidum* among men who have sex with men [25–27], low evidence is found among heterosexual couples [22]. Indeed, this is one of the first investigations to report an association of anal sex between men and women and syphilis infection. This result indicates that not only the use of condoms is important to the spread of STIs, but also the type of sexual practice among heterosexual couples. Thus, more studies are required to clarify the risk of anal intercourse on syphilis transmission in heterosexual sex that was found on this investigation.

Infectious transmissible diseases are a threat not only for the more vulnerable marginalised populations but can spread to an entire society, especially when associated with sexual activities. Controlling the infection in populations at risk also helps to control the spread of diseases in the entire population. Thus, public health institutions should perform several actions with key populations such as (i) educational program to present and discuss the risks involved in sexual practices; (ii) offering rapid syphilis tests and possibilities of self-testing (iii); stimulating public awareness about syphilis transmission and disease signs and symptoms (iv); continually offer treatment and reinforces treatment adherence; (v) conduct regular vaccination campaigns against other associated pathogens such as HBV and HPV; (vi) regularly offer methods for sexual protection, such as condoms and lubricants for anal sex.

Sex work is usually a matter of need, not of choice. So, governments and other human organizations can help risk groups, such as FSWs, by creating ways to promote self-esteem, self-confidence, and stimulate awareness of social rights and benefits, considering their vulnerability that place them in a group of risk, and also alerting them how they can be a key player in disease spread/control [13, 14]. As an example, a comprehensive health program was planned and implemented with FSW in southern China, resulting in several public health benefits, including in the prevention and control of syphilis [28].

This study has some limitations. Firstly, limited sample size and restriction to three cities, which makes the results not necessarily representative of the FSW population of the state of Pará, Brazil. Another limitation is that the diagnosis was exclusively based on serological tests, no clinical examinations were performed in order to check signs of active disease. Also, recently acquired infections can present a small concentration of antigens and antibodies that aren’t detected yet by either VDRL and ELISA and therefore may have been diagnosed as negative. In addition, although convenience sampling has been found to be adequate for quasi-representative sampling in hidden populations, other sampling methods could have been used to improve representativeness. Finally, bias is always a risk in data from self-reported forms and the cross-sectional design of this study limits its capacity to establish causality.

## Conclusions

This study has identified a high prevalence of syphilis among FSWs in the Brazilian Amazon region, which is 15 times higher than observed in a general population and showing that *T. pallidum* infections are more likely to be transmitted FSWs in this particular region when compared to other large and higher-income Brazilian cities. Low level of education was a factor associated with syphilis infection, a condition that can be found in several other populations worldwide. Anal intercourse was identified as a risk factor among heterosexual couples, a situation previously associated mainly with men who have sex with men. Comprehensive health programs targeting vulnerable groups, considering their characteristics and peculiarities, can be a rational way to reduce syphilis spread, which constitutes a major rising public health problem in Brazil and in several other countries.

## Data Availability

The datasets analyzed during the current study are not publicly available due to the progress of analyzes of possible infections and co-infections with other pathogens, but are available from the corresponding author on reasonable request.

## Abbreviations

EDTA: Ethylenediaminetetraacetic acid
ELISA: Enzyme-linked immunosorbent assay
FSWs: Female sex workers
HIV: Human immunodeficiency virus
RP: Reasons of prevalence
STIs: Sexually transmitted infections
VDRL: Venereal disease research laboratory
WHO: World Health Organization
